# Construction of a Stable Replicating Shuttle Vector for *Caldicellulosiruptor* Species: Use for Extending Genetic Methodologies to Other Members of This Genus

**DOI:** 10.1371/journal.pone.0062881

**Published:** 2013-05-03

**Authors:** Daehwan Chung, Minseok Cha, Joel Farkas, Janet Westpheling

**Affiliations:** 1 Department of Genetics, University of Georgia, Athens, Georgia, United States of America; 2 The BioEnergy Science Center, Department of Energy, Oak Ridge National Laboratory, Oak Ridge, Tennessee, United States of America; Belgian Nuclear Research Centre SCK/CEN, Belgium

## Abstract

The recalcitrance of plant biomass is the most important barrier to its economic conversion by microbes to products of interest. Thermophiles have special advantages for biomass conversion and members of the genus *Caldicellulosiruptor* are the most thermophilic cellulolytic microbes known. In this study, we report the construction of a replicating shuttle vector for *Caldicellulosiruptor species* based on pBAS2, the smaller of two native *C. bescii* plasmids. The entire plasmid was cloned into an *E. coli* cloning vector containing a pSC101 origin of replication and an apramycin resistance cassette for selection in *E. coli*. The wild-type *C. bescii pyrF* locus was cloned under the transcriptional control of the regulatory region of the ribosomal protein S30EA (Cbes2105), and the resulting vector was transformed into a new spontaneous deletion mutant in the *pyrFA* locus of *C. bescii* that allowed complementation with the *pyrF* gene alone. Plasmid DNA was methylated *in vitro* with a recently described cognate methyltransferase, M.CbeI, and transformants were selected for uracil prototrophy. The plasmid was stably maintained in low copy with selection but rapidly lost without selection. There was no evidence of DNA rearrangement during transformation and replication in *C. bescii*. A similar approach was used to screen for transformability of other members of this genus using M.CbeI to overcome restriction as a barrier and was successful for transformation of *C. hydrothermalis,* an attractive species for many applications. Plasmids containing a carbohydrate binding domain (CBM) and linker region from the *C. bescii celA* gene were maintained with selection and were structurally stable through transformation and replication in *C. bescii* and *E. coli*.

## Introduction

The recalcitrance of plant biomass is an obstacle to the efficient production of biofuels and bioproducts from renewable plant sources [1]–[3]. It is also unclear which types of biomass will be most economical as substrates and it is likely that more than one type will be needed. In addition, traditional pretreatments to overcome plant biomass recalcitrance often involve harsh chemical and thermal treatments [4], [5], or enzymatic digestion which are expensive [6]. Thermophilic bacteria have advantages for use in biomass conversion and members of the genus *Caldicellulosiruptor*, are the most thermophilic cellulolytic microbes known and have the ability to grow on non-pretreated lignocellulosic biomass [7]–[9]. The sequences of eight *Caldicellulosiruptor* genomes have been published [10]–[15] and microarray analysis of cells grown on various substrates implicates genes encoding multi-domain, multi-functional CAZy (Carbohydrate Active Enzymes) proteins organized into large clusters predicted to act synergistically in biomass degradation [16]–[18]. Interestingly, different species have different growth characteristics depending on the biomass and some species have substantial differences in the use of crystalline cellulose [7], [9], [16]. We recently reported an efficient method for DNA transformation of *C. bescii* that relies on methylation of transforming DNA with an endogenous α-class N4-cytosine methyltransferase, M.CbeI [19]. The ability to genetically manipulate all members of this genus will be important to realize their potential for Consolidated BioProcessing (CBP) [20].

Replicating shuttle vectors facilitate a variety of genetic manipulations including optimization of transformation protocols to establish reliable genetic systems and homologous and heterologous expression of genes of interest. For members of the *Caldicellulosiruptor* genus, they will be an important tool for extending substrate utilization for plant biomass deconstruction and metabolic engineering pathways for the production of biofuels and bioproducts. Several shuttle vectors, pIKMI in *Thermoanaerobacter* JW/SL-YS485 [21], pCTCI in *Clostridium cellulolyticum* ATCC 35319 [22], pUB110 in *Bacillus stearothermophilus* [23], pMU612 in *Clostridium thermocellum* [24], and pHV33 and pMK419 in *Clostridium thermocellum* [25], have been successfully used to transform anaerobic thermophilic bacteria. Most reported shuttle vectors rely on drug resistance markers for selection but these are not suitable for use in hyperthermophiles like *Caldicellulosiruptor* species which grow optimally at and above 70°C because either the antibiotics themselves or the corresponding resistance gene products are not stable at 75°C. We recently reported the use of a deletion in the *pyrBCF* locus of *C. bescii* for nutritional selection of transformants [19].

**Figure 1:**
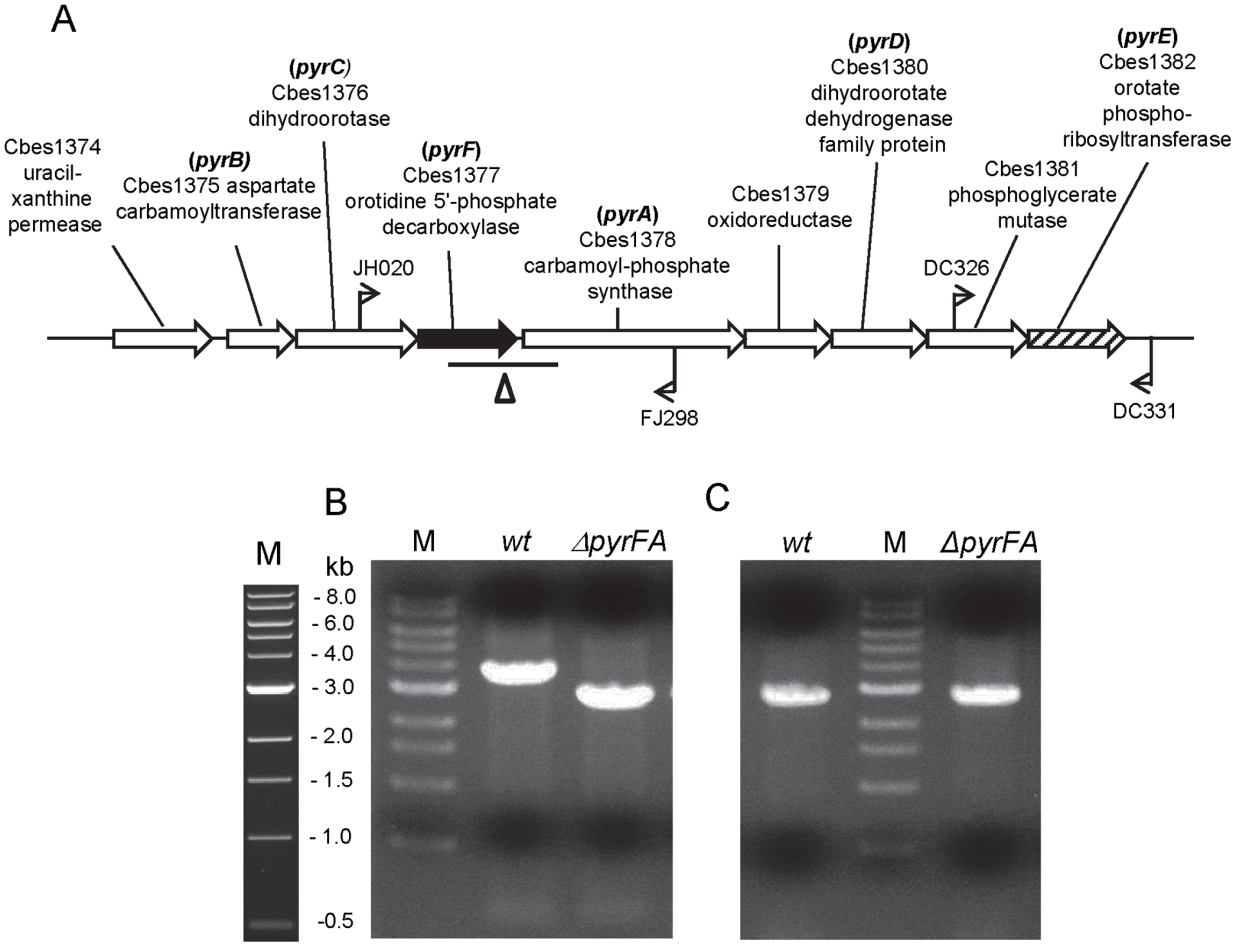
Chromosomal map and PCR analysis of the Uridine Monophosphate (UMP) biosynthetic gene cluster in *C.bescii* DSM 6725 and the spontaneous deletion in *pyrFA* (JWCB005) locus. (A) A diagram of the *pyr* operon region with the 878 bp deletion in the *pyrFA* ORFs. The line below the diagram indicates the length of the deletion. Bent arrows depict primers used for verification of the structure of the chromosome in the JWCB005 (Δ*pyrFA*) strain. *pyrF* and *pyrE* loci indicated as black color filled arrow and black dashed filled arrow, respectively. (B) Gel depicting PCR products of the *pyrFA* region in wild type (3.44 kb) compared to the Δ*pyrFA* (2.52 kb) strain amplified by primers (JH020 and FJ298). (C) Gel depicting the 2.66 kb PCR products of *pyrE* region in wild type and the Δ*pyrFA* strain by primers (DC326 and DC331). M: 1 kb DNA ladder (NEB).

Here we report the isolation and characterization of new uracil auxotrophic mutant of *C. bescii*, JWCB005, that contains a deletion in the *pyrFA* locus (Fig. 1A, Table 1) and allows complementation by the *pyrF* gene alone. We constructed a replicating shuttle vector based on the smaller of two native *C. bescii* plasmids, pBAS2 [17], [26], that contains the wild type *pyrF* allele for uracil prototrophic selection. This plasmid is capable of stable replication and selection in both *E. coli* and *C. bescii*. Plasmid DNA was unchanged during transformation and replication in *C. bescii* and back transformation into *E. coli*. Transformation with replicating plasmid DNA is an order of magnitude more efficient than transformation with non-replicating plasmids in *C. bescii* [19], making this vector an important tool to screen transformability of members of this genus. Using similar methods as for *C. bescii*, we were able to use this plasmid to transform *C. hydrothermalis*. In addition we demonstrated the utility of this vector as a cloning vector by cloning a portion of the *celA* gene from *C. bescii*.

**Table 1:** Strains and plasmids used in this work.

Strains or plasmid	Strain and genotype/phenotype	Source
*Caldicellulosiruptor*		
JWCB001	*C. bescii* DSM6725 wild type (*ura* ^+^/5-FOA^S^)	DSMZ^1^
JWCB005	*C. bescii ΔpyrFA* (*ura* ^−/^5-FOA^R^)	This study
JWCB011	*C. bescii* JWCB005 transformed with pDCW89 (*ura* ^+^/5-FOA^S^)	This study
JWCB014	*C. bescii* JWCB005 transformed with pDCW129 (*ura* ^+^/5-FOA^S^)	This study
JWCH003	*C. hydrothermalis* IS*CahyI* insertion mutation in *pyrF* (*ura* ^−/^5-FOA^R^)	[35]
JWCH005	JWCH003 transformed with pDCW89 (*ura* ^+^/5-FOA^S^)	This study
*Escherichia coli*		
JW261	DH5α containing pDCW68 (Apramycin^R^)	[30]
JW292	DH5α containing pDCW89 (Apramycin^R^)	This study
JW301	DH5α containing pDCW129 (Apramycin^R^)	This study
JW319	DH5α containing pDCW154 (Apramycin^R^)	This study
JW320	DH5α containing pDCW155 (Apramycin^R^)	This study
Plasmids		
pDCW68	6–8 copy plasmid DNA (Apramycin^R^)	[30]
pDCW89	*E. coli/Caldicellulosiruptor* species shuttle vector (Apramycin^R^)	This study
pDCW129	*E. coli/Caldicellulosiruptor* species shuttle vector (Apramycin^R^)	This study
pDCW154	6–8 copy plasmid DNA (Apramycin^R^)	This study
pDCW155	6–8 copy plasmid DNA (Apramycin^R^)	This study

1 German Collection of Microorganisms and Cell Cultures.

## Materials and Methods

### Strains, Media and Growth Conditions

*C. bescii*, *C. hydrothermalis*, and *E. coli* strains used in this study are listed in Table 1. *Caldicellulosiruptor* species were grown anaerobically in liquid or solid modified DSMZ516 medium [19] or in low osmolarity defined (LOD) growth medium [27] with maltose as the sole carbon source as described at 75°C for *C. bescii* or at 68°C for *C. hydrothermalis*. For growth of auxotrophic mutants JWCB005 and JWCH003, the defined medium containing 40 µM uracil was used. *E. coli* strains DH5α (*dam^+^ dcm^+^*), BL21 (*dam^+^ dcm^−^*), and ET12567 (*dam^−^ dcm^−^)* were used for plasmid DNA constructions and preparations. Standard techniques for *E. coli* were performed as described [28]. *E. coli* cells were grown in L broth supplemented with apramycin (50 µg/mL), kanamycin (25 µg/mL), or spectinomycin (20 µg/mL), where appropriate. *E. coli* plasmid DNA was isolated using a Qiagen Mini-prep Kit. Chromosomal DNA from *Caldicellulosiruptor* species was extracted using the Quick-gDNA™ MiniPrep (Zymo) according to the manufacturer’s instructions. Total DNA was isolated from *Caldicellulosiruptor* species as described [29], except that adding additional lysozyme (30 µg/mL) for 1 hour at room temperature in lysis buffer [30] and sonication were employed to enhance the cell lysis. Plasmid DNA isolation from *Caldicellulosiruptor* species was performed as described [30].

### Isolation of 5-FOA resistant/uracil Auxotrophic Mutants

A spontaneous deletion within the *C. bescii* DSM6725 *pyrFA* locus (Fig. 1A, Table 1) was isolated using the same approaches as previously described [19]. Growth of this strain, JWCB005, supplemented with uracil (40 µM) was comparable to wild type reaching a cell density of ∼2×10^8^ in 24 hours. Cells were counted in a Petroff-Housser counting chamber using a phase-contrast microscope with 40X magnification.

### Construction of Plasmids

**Figure 2:**
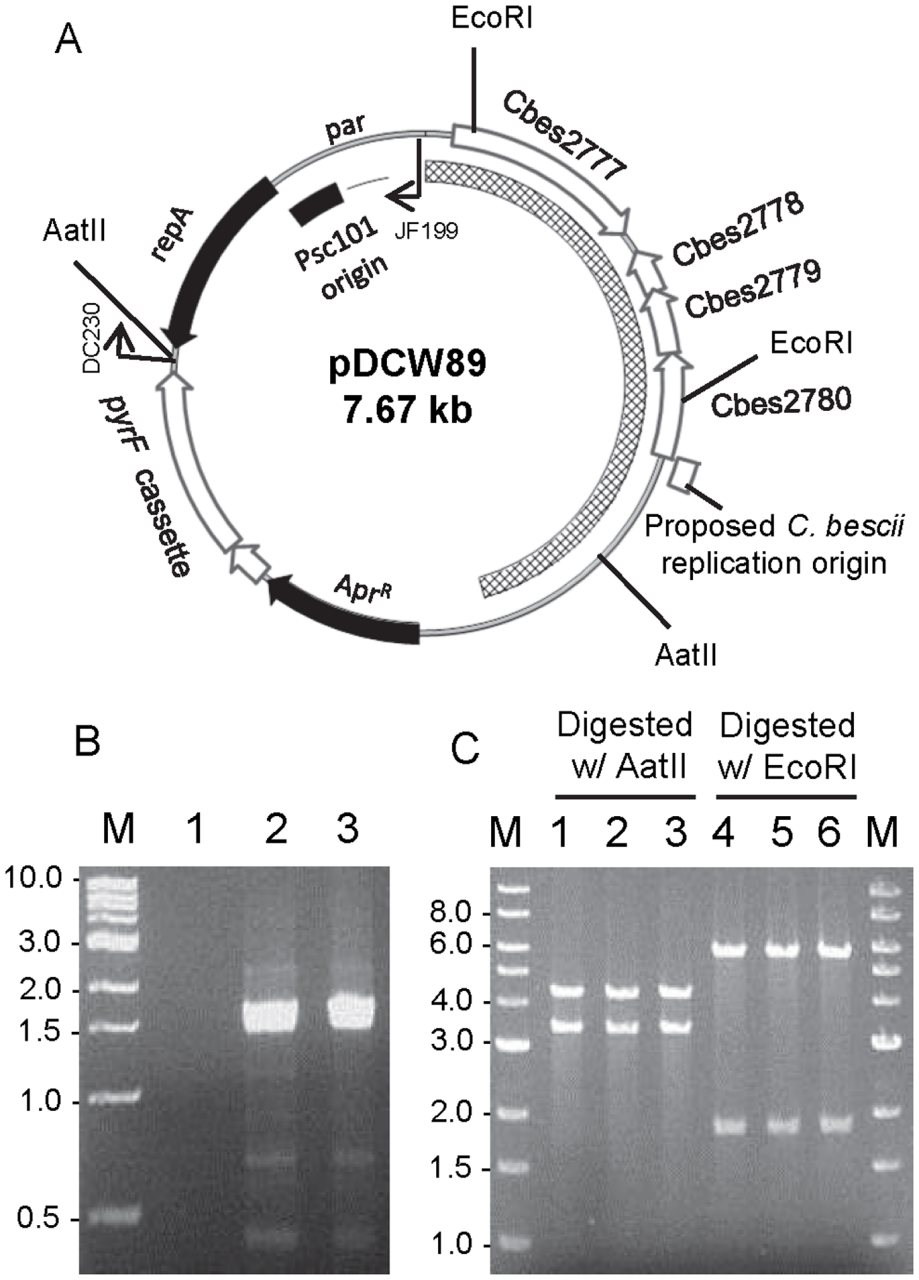
Plasmid map of shuttle vector (pDCW89) and verification of its presence in *C.bescii* transformants. (A) A linear DNA fragment containing the *pyrF* expression cassette as well as the entire sequence of pBAS2, generated by PCR amplification using primers DC283 and DC284, was ligated to a DNA fragment containing *E. coli* replication and selection functions to generate the final shuttle vector. The cross-hatched box corresponds to the pBAS2 plasmid sequences. ORFs from *C. bescii* are indicated as empty arrows and those from *E. coli* as black arrows. The apramycin resistant gene cassette (Apr^R^); PSC101 low copy replication origin in *E. coli*; *repA*, a plasmid-encoded gene required for PSC101 replication; *par*, partition locus are indicated. The proposed replication origin (115 bp) of pBAS2 is indicated. The primers and restriction sites (AatII and EcoRI) used for the verification are indicated. A detailed description of the construction of pDCW89 is described in Fig. S1 and the Materials and Methods. (B) Gel showing the 1.6 kb PCR products containing the pSC101 *ori* sequences only presence in pDCW89 using primers DC230 and JF199, total DNA from JWCB005 (Lane 1), a *C. bescii* transformant with pDCW89 (Lane 2), and pDCW89 isolated from *E. coli* (Lane 3) as template. (C) Restriction analysis of plasmid DNA before and after transformation of *C. bescii* and back-transformation to *E. coli*. Lanes 1 and 4, pDCW89 plasmid DNA isolated from *E. coli* DH5α, and digested with AatII (Lane 1, 4.4 kb and 3.3 kb cleavage products), and EcoRI (Lane 4, 1.9 kb and 5.8 kb cleavage products); lane 2, 3, 5, 6, plasmid DNA isolated from two biologically independent *E. coli* DH5α back-transformed from *C. bescii* transformants, and digested with AatII (Lane 2 & 3), and EcoRI (Lane 5 & 6). M: 1 kb DNA ladder (NEB).

Plasmids were generated using high fidelity *pfu AD* DNA polymerase (Agilent Technologies), restriction enzymes (NEB), and Fast-link™ DNA Ligase (Epicentre Technologies) according to the manufacturer’s instructions. Plasmid pDCW89 (Fig. 2A, Fig. S1) was constructed in three cloning steps. First, a 2.9 kb of DNA fragment, containing the PSC101 replication origin and an apramycin resistance gene cassette (Apr^R^), was amplified by PCR using the primers DC080 and DC084 and pDCW68 [30] as template. The 2.9 kb DNA fragment was then blunt-end ligated after treatment with T4 PNK (NEB) to construct Intermediate vector I (Fig. S1). A cassette containing the wild-type *pyrF* gene was constructed by overlap extension polymerase chain reaction (OE-PCR) placing the *pyrF* gene under the transcriptional control of the ribosomal protein S30EA (Cbes2105) promoter. A 199 bp portion of the regulatory region of Cbes2105 was amplified from wild-type genomic DNA using primers DC175 and DC174. The *pyrF* (Cbes1377, 918 bp) gene was amplified using primers DC173 and DC232, and joined to the fragment containing the regulatory region by OE-PCR. The DC175 and DC232 primers were engineered to contain NheI and AatII sites, respectively. The 2.9 kb DNA fragment were amplified by PCR from intermediate vector 1 using primers DC176 and DC230 to add restriction sites. The DC176 and DC230 primers were also engineered to contain NheI and AatII sites, respectively. The two linear DNA fragments were digested with NheI and AatII, and ligated to generate 4.02 kb size of intermediate vector II (Fig. S1). In the last step, a 3.65 kb DNA fragment containing the entire sequence of pBAS2 [17], [26] were amplified by PCR using DC283 and DC284, that contained restriction sites added a KpnI site at 5′ end and a XhoI site at 3′ end. A 4.02 kb linear fragment was amplified from intermediate vector II using DC285 and DC286, which contain engineered restriction sites, KpnI and XhoI, respectively. The two linear DNA fragments were digested with KpnI and XhoI, and ligated to generate pDCW 89. Further details of this construction are described in Fig. S1. Plasmid pDCW129 was generated inserting a 0.68 kb DNA fragment containing the carbohydrate binding domain (CBM) and linker region derived from the *celA* gene (Cbes1867) into pDCW89. A 0.68 kb DNA fragment was amplified by PCR using the DC397 and DC398 primers and total DNA isolated from *C. bescii* as template. The 7.75 kb of backbone DNA fragment was amplified by PCR using the DC365 and DC399 and pDCW89 as template. DC397 and DC365 primers were engineered to contain a BamHI site at the end. DC398 and DC399 primers were engineered to contain a SphI site at the end. The two linear DNA fragments were digested with BamHI and SphI and ligated to generate pDCW129. Plasmid pDCW154 and pDCW155 were generated to reduce the size of pDCW89 (Fig. S3). The 4.02 kb backbone DNA fragment was amplified by PCR using primers DC081 and DC507 and pDCW89 as template. The DC081 and DC507 primers were engineered to contain KpnI and PvuII sites, respectively. To generate pDCW154, a 531 bp DNA fragment derived from pBAS2 [17], [26] was amplified by PCR using DC505 and DC506, primers with engineered restriction sites, KpnI and PvuII, respectively. Two linear DNA fragments were digested with KpnI and PvuII, and ligated to generate pDCW154 (Fig. S3A). Plasmid pDCW155 (Fig. S3B) is identical to pDCW154 except that the 851 bp DNA fragment was replaced with the 531 bp of fragment. The 851 bp DNA fragment derived from pBAS2 [17], [26] was amplified by PCR using DC508 and DC 506, primers with engineered restriction sites, KpnI and PvuII, respectively. This 851 bp DNA fragment was subsequently ligated into a 4.02 kb backbone DNA fragment as described for pDCW154. DNA sequences of the primers are shown in Table S1. *E. coli* strain DH5α cells were transformed by electroporation in a 2-mm-gap cuvette at 2.5 V and transformants were selected for apramycin resistance. The sequences of all plasmids were confirmed by Automatic sequencing (Macrogen USA, Maryland). All plasmids are available on request.

### Transformation of *Caldicellulosiruptor* Species

Electrotransformation of JWCB005 and JWCH003 was performed as described [19]. JWCB011 and JWCB014 were generated by transforming JWCB005 with M.CbeI methylated pDCW89 and/or pDCW129 as described and selecting for uracil prototrophy at 75°C. DNA transformation of *C. bescii* was confirmed by PCR analysis using primers DC230 and JF199 or primers DC233 and DC235, and also by back-transformation to *E. coli*. Transformation of the JWCH003 strain was performed similarly, but at 68°C. Transformation of pDCW89 into *C. hydrothermalis* was confirmed by direct plasmid DNA isolation from transformant, JWCH005. The transformation efficiencies were calculated as the number of transformant colonies per µg of DNA added and do not take into account plating efficiencies. *E. coli* strain DH5α cells were transformed by electroporation in a 2 mm gap cuvette at 2.5 V, and transformants were selected for apramycin resistance.

### Assessment of Relative Copy Number, Maintenance and Stability

*C. bescii* transformants (JWCB011) were serially subcultured every 16 hours for 5 passages in selective (without Uracil) and non-selective (supplemented with 40 µM uracil) liquid media. After each passage, cells were harvested and used to isolate total DNA. For each sample, 3 µg of total DNA was digested with 10 U of EcoRV for 6 hours at 37°C. The restriction fragments were separated by electrophoresis in a 1.0% (wt/vol) agarose gel and transferred onto nylon membranes (Roche). Primers JF396 and JF397 were used to amplify a fragment of the *pyrF* gene using JWCB005 genomic DNA as template to generate a digoxigenin (DIG)-labeled probe by random priming with DIG High Prime DNA Labeling and Detection Starter Kit I (Roche). The membrane was incubated with probe at 42°C and washed at 65°C. Band intensities were determined by using a Storm 840 PhospoImager (GE Healthcare) equipped with ImageQuant v.5.4 software (Molecular Dynamics). Relative copy number was determined as the ratio of band intensity of the plasmid derived band to the chromosomal *pyrF* fragment. Plasmid maintenance with and without selection was inferred from the change in relative copy number over the 5 successive cultures. To assess the structural stability of the plasmid, total DNA isolated from five independent *C. bescii* transformants containing pDCW89 was used to back-transform *E. coli* for plasmid isolation and restriction digestion analysis.

### Determine the Relative Copy-number of pBAS2

Total DNA was isolated from JWCB001 (Table 1) and treated with RNase A (Qiagen). qPCR experiments were carried out with an LightCycler 480 Real-Time PCR instrument (Roche) with a LightCycler 480 SYBR Green I master mix (Roche). The relative copy-number of pBAS 2 [17], [26] was determined as the average of two biologically independent samples. Table S1 lists the primers used in the qPCR experiment.

## Results

### Isolation of a Spontaneous Deletion of the *C. bescii pyrFA* Locus for Nutritional Selection of Transformants

As we reported previously [19], [30], attempts to use drug resistance markers for selection of transformants in *C. bescii* were unsuccessful either because the gene products were unstable at 75°C or because of high levels of natural resistance in *C. bescii*. Orotidine monophosphate (OMP) decarboxylase, encoded by the *pyrF* gene in bacteria (*ura3* in yeast), converts the pyrimidine analog 5-fluoroorotic acid (5-FOA) to 5-fluorouridine monophosphate which is ultimately converted to fluorodeoxyuridine by the uracil biosynthetic pathway, a toxic product that kills growing cells that are synthesizing uracil [31]. Mutants of *pyrF* are, therefore, uracil auxotrophs and resistant to 5-FOA, providing uracil prototrophy as a selection for the wild type allele and 5-FOA resistance as a counter selection for the mutant allele. We previously reported the isolation and use of such a mutant for nutritional selection of transformants, but as it contained a deletion of most of the *pyrBCF* region complementation required all three genes [19]. Cloning a large region of the chromosome for complementation was not optimal for designing a replicating plasmid vector since increased size often results in lower transformation efficiency. We isolated a different deletion mutant, in the *pyrFA* locus, that was complemented by the *pyrF* gene alone and it was used in this study.

To obtain this new deletion strain, *C. bescii* cells were plated on modified DSMZ 640 media [19] containing 8 mM 5-FOA. Spontaneous resistance to 5-FOA was observed at a frequency of approximately 10^−5^ at 65°C. Among 30 mutants isolated, one, designated JWCB005 (Table 1), had an 878 bp deletion that spans most of the *pyrF* open reading frame (Cbes1377), and part of the adjacent gene, *pyrA* (Cbes1378) (Fig. 1A). The extent of the deletion was defined by PCR amplification of the *pyrFA* region in the mutant and subsequent sequencing of the PCR product (Fig. 1B). We also PCR amplified and sequenced the *pyrE* region, also required for uracil biosynthesis, and found it to be wild type (Fig. 1C). JWCB005 was a tight uracil auxotroph capable of growth in media supplemented with uracil, but not orotate, confirming that *pyrF* function was absent in this deletion. The function of *pyrA* does not seem to be affected by the deletion, because transformation with pDCW89, containing only the wild type *pyrF* allele, was able to complement the uracil auxotrophy without added orotate, the product of *pyrA* in uracil biosynthetic pathway. As with all such deletions, reversion to uracil prototrophy was not a concern making prototrophic selection possible no matter how low the frequency of transformation. Growth of this mutant (JWCB005) supplemented with uracil (40 µM) was comparable to that of the wild type, reaching a cell density of ∼2×10^8^ in 24 hours.

### Construction of a Replicating Shuttle Vector Based on pBAS2

*C. bescii* contains two native plasmids, pBAL and pBAS2, 8.3 kb and 3.7 kb, respectively [17], [26]. Because of its relatively small size, we chose to use pBAS2 to supply replication functions for *C. bescii* in the shuttle vector. The pBAS2 plasmid was first reported nearly a decade ago, but the copy number was never determined. The plasmid contains sequences that show homology to a double stranded replication origin, characteristic of plasmids with rolling circle replication, however no single stranded intermediates of plasmid replication were detected [26]. The plasmid encodes 10 putative open reading frames, 4 of which are predicted to be genes [17], [26]. The replication mechanism of pBAS2 was also supported by pylogenetic analysis. The 149 amino acid protein Cbes2780 shares 30% sequence identity to the 142 amino acid rolling circle replication protein from *Bacillus thuringiensis serovar sotto str.* sp [32] and ∼43% similarity to the putative rolling circle replication protein from *Pseudoalteromonas sp. PS1M3* [26], [33]. It also shares weak sequence identity to a plasmid replication protein from *Pseudomonas savastanoi* (25%) and *Paracoccus marcusii* (24%). plasmid replication protein, respectively [26]. To avoid disrupting the replication functions of the pBAS2 plasmid, we linearized the plasmid DNA just upstream of the Cbes2777 ORF and inserted the *aac* gene for selection of apramycin resistance in *E. coli*. The *C. bescii pyrF* gene, under the transcriptional control of the promoter of the ribosomal protein S30EA (Cbes2105), was used for selection of uracil prototrophy in the *C. bescii pyrFA* deletion mutant (JWCB005), and the pSC101 replication origin for replication in *E. coli*. The resulting plasmid, pDCW89 (Fig. 2A), was transformed into *C. bescii* by electroporation, and cells were plated onto defined medium without uracil as described [19]. Uracil prototrophic colonies were selected and transformation was confirmed by PCR amplification of a portion of the pSC101 replication origin present only in pDCW89 (Fig. 2B). Total DNA isolated from two biologically independent transformants was used to back-transform *E. coli*. Restriction digestion analysis showed that the plasmid was unchanged during transformation and replication in *C. bescii* and/or subsequent back transformation to *E. coli* (Fig. 2C). This result suggests that it was replicating autonomously in both organisms. The resulting strain was designated JWCB011 (Table 1). The transformation frequency varied between experiments, but was typically about 500 transformants per µg of plasmid DNA. This efficiency was 10 times higher than the transformation efficiency observed with non-replicating plasmids in *C. bescii* [19].

### Assessment of Plasmid Maintenance, and Relative Copy Number in *C. bescii*

To assess plasmid maintenance and relative copy number, *C. bescii* transformants were serially sub-cultured every 16 hours for 5 passages in selective and nonselective liquid LOD medium [27]. Total DNA isolated from cells after each passage was used for Southern hybridization analysis (Fig. S2). To generate a probe for the detection of a sequence contained once on both the plasmid and the chromosome, primers JF396 and JF397 were used to amplify a fragment of the *pyrF* gene remaining in the genome of JWCB005, and also contained on the plasmid. Relative copy number was determined as the ratio of band intensity of the plasmid derived copy of the *pyrF* locus (7.7 kb) compared to the chromosomal derived copy of the *pyrF* locus (3.7 kb) in JWCB005 (Fig. S2). The relative intensity was 0.8 to 1.1 suggesting that the shuttle vector exists as a single copy per chromosome (Fig. S2). Most plasmids that replicate via a rolling circle mechanism exist in high copy per chromosome [34] and the native pBAS2 may exist in high copy as well. The relative copy-number of pBAS2 was determined by qPCR with primer pairs targeting specific regions of pBAS2 and/or the chromosome. The relative copy-number of pBAS2 was calculated to be seventy-five copies per chromosome based on two biologically independent analyses. The fact that the shuttle vector exists in a single copy per chromosome likely reflects the fact that it is competing with the endogenous replicon as they share replication and maintenance functions. The 4.3 kb band indicates the *pyrF* containing fragment in wild type *C. bescii* (lane 12) and 8.3 kb band is non-specific hybridization with pBAL, the larger of two endogenous plasmids in *C. bescii* (Fig. S2).

Plasmid maintenance was determined by assessing the presence of the plasmid after passage with and without nutritional selection over the 5 successive transfers. Southern analysis showed that the plasmid relative copy number remains constant with selection, but that the plasmid is quickly lost without selection (Fig. S2). A single passage in nonselective media (with 40 µM uracil) is enough for the plasmid to be lost from the majority of cells (Fig. S2). Both the very low copy number and rapid loss without selection may be due to plasmid incompatibility between pBAS2 and pDCW89. Efforts are underway to cure the resident plasmid but there are advantages to having a shuttle vector that may be cured easily.

### Transformation of *C. hydrothermalis* with Shuttle Vector DNA Methylated with M.CbeI

**Figure 3:**
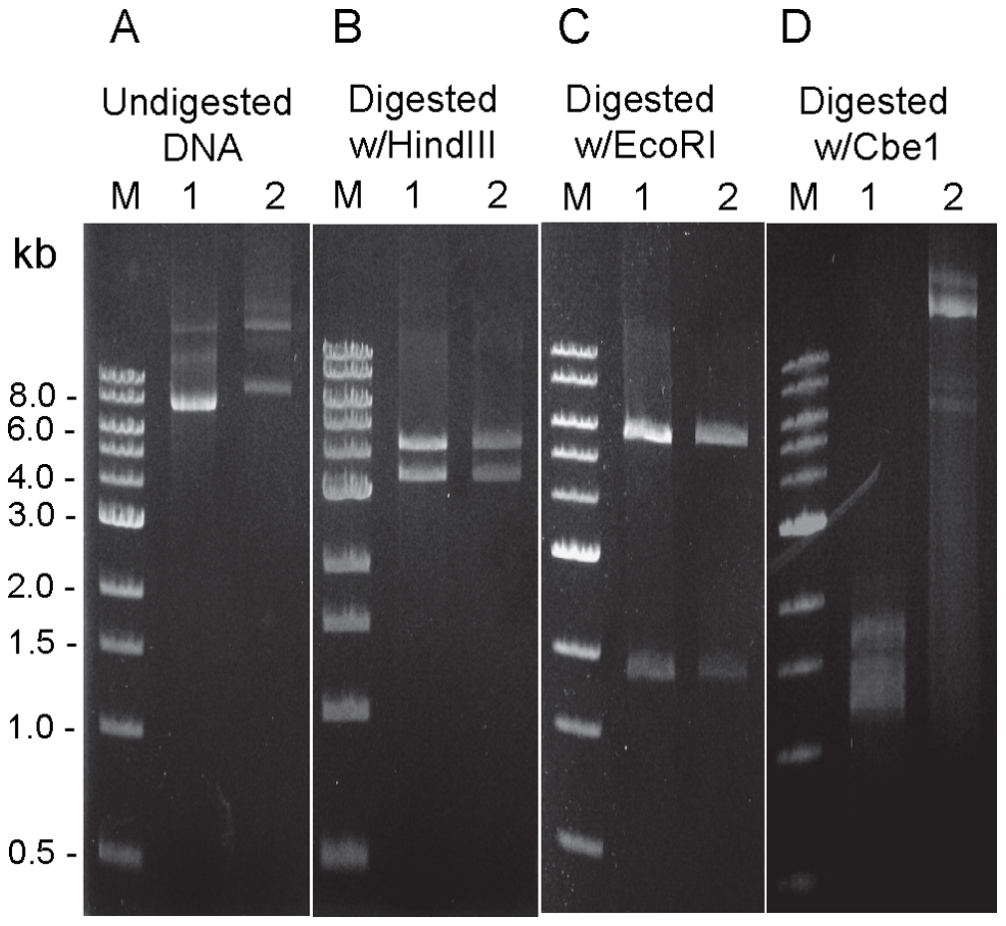
Comparison of DNA modification status between shuttle vector DNA isolated from *E.coli* (Lane 1) and *C. hydrothermalis* transformants (Lane 2) by Restriction analysis. (A) Undigested, (B) Digested with HindIII (4.3 and 3.4 kb cleavage products); (C) Digested with EcoRI (4.6 and 1.9 kb cleavage products); (D) Digested with CbeI (11 cleavage products are expected). M: 1 kb DNA ladder (NEB).

Restriction of transforming DNA is a major and, for *C. bescii*, apparently an absolute barrier to transformation of DNA from *E. coli* [19], [30]. Transformation of plasmid DNA from *E. coli* into *C. bescii* requires *in vitro* methylation with an endogenous α-class N4-Cytosine methyltransferase, M.CbeI [19]. To test whether modification by M.CbeI also allowed transformation of other members of this genus, a spontaneous mutation resistant to 5-FOA was isolated in *C. hydrothermalis* [35], JWCH003 (Table 1). This mutant was a tight uracil auxotroph and was used as a host for plasmid transformation. Unmethylated plasmid DNA isolated from various *E. coli* hosts failed to transform this mutant but DNA that has been methylated with M.CbeI transformed at a frequency similar to that for *C. bescii* (typically about 500 transformants per µg of plasmid DNA). Transformants were initially confirmed by PCR amplification of the *aac* gene contained exclusively on the plasmid (data not shown). As shown in Fig. 3, restriction digestion analysis using HindIII and EcoRI of shuttle vector plasmid DNA isolated from *C. hydrothermalis* transformants was indistinguishable from that isolated from *E. coli* (Fig. 3B and 3C) suggesting that is structurally stable in *C. hydrothermalis*. This further suggests that modification with M.CbeI may have utility in DNA transformation of a variety of *Caldicellulosiruptor* species and current efforts to isolate deletions of the *pyrF* locus in a number of other species to test this are in progress. These data also provide evidence that the use of the wild type *C. bescii pyrF* allele under the control of the ribosomal protein S30EA (Cbes2105) promoter functions in at least one other species and will likely prove to be a useful selection marker for many species. We anticipate that this shuttle vector will facilitate extension of genetic methods to a number of other *Caldicellulosiruptor* species.

While shuttle vector plasmid DNA was difficult to isolate from *C. bescii*, requiring back transformation into *E. coli* for analysis, it was readily isolated from *C. hydrothermalis* suggesting that the vector may exist in higher copy in *C. hydrothermalis* as there is no competing plasmid present in this species. The relatively copy number of pDCW89 in *C. hydrothermalis* was difficult to determine because the spontaneous mutation to 5-FOA resistance (a uracil auxotrophy) reverted at high frequency. Subsequent analysis of this mutant revealed that the mutation resulted from a transposon (IS*Cahy1*) insertion into the *pyrF* locus of *C. hydrothermalis* [35]. The amount of isolated plasmid DNA varied widely without selection. Attempts are underway to isolate a deletion of the *pyrF* locus in *C. hydrothermalis*, which would eliminate the issue of the reversion to uracil prototrophy. We point out that, as shown in Fig. 3A, the uncut plasmid DNA isolated from *C. hydrothermalis* migrated slower than that isolated from *E. coli*. This may be due to differences in the degree of methylation of the DNA, in these different hosts. These results suggest that *C. hydrothermalis*, like *C. bescii,* contains a functional CbeI/M.CbeI like restriction-modification system [19], [30]. In support of this is the fact that, pDCW89 isolated from *C. hydrothermalis* was resistant to digestion by purified CbeI or HaeIII (NEB) endonucleases (Fig. 3D).

### Cloning of a CBM and a Linker Region of the *celA* Gene into pDCW89

**Figure 4:**
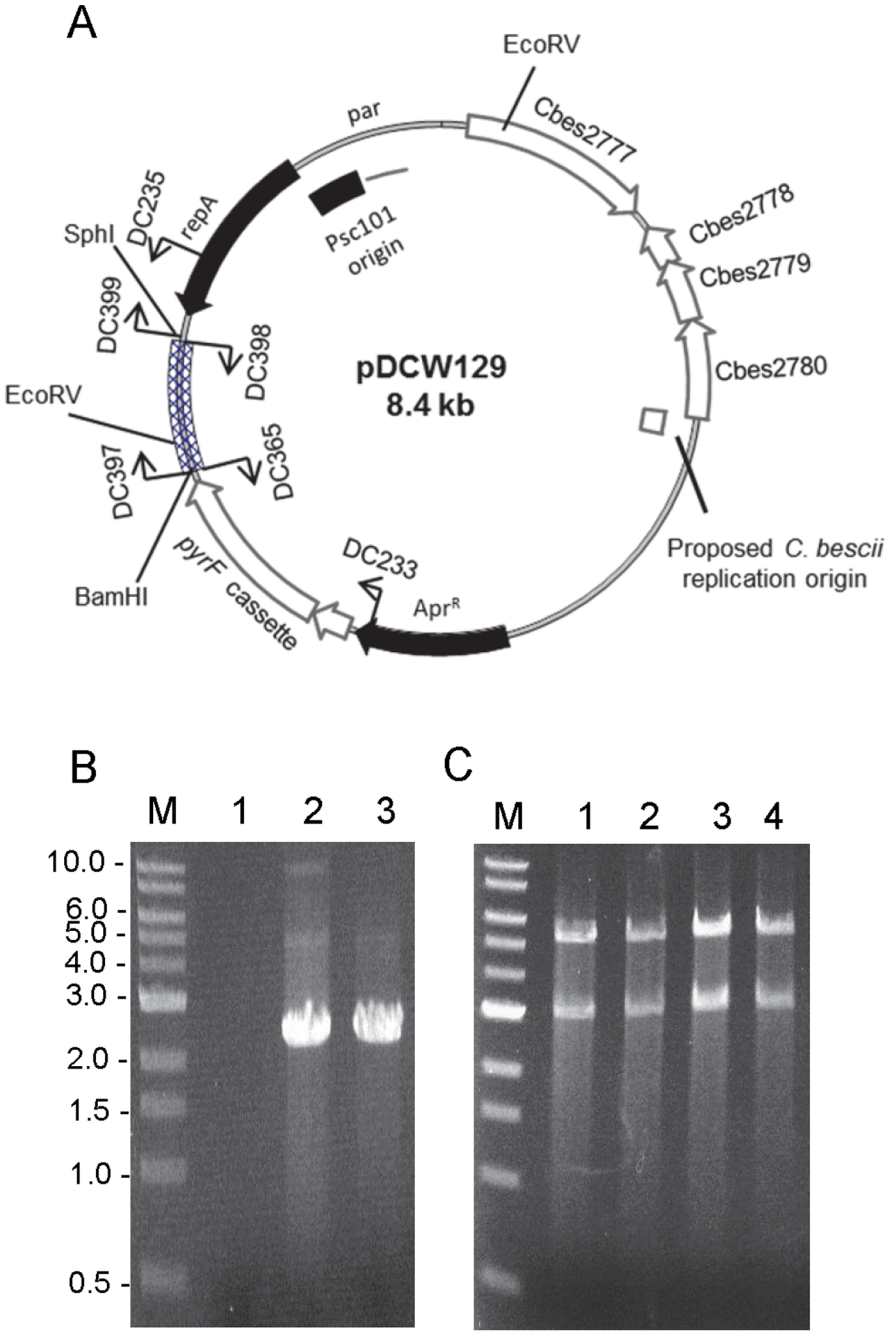
Plasmid map of pDCW129 and verification of its ability to structurally stable maintenance of inserted DNA fragment through transformation and replication in *C.bescii*. (A) Diagram of pDCW129. A linear DNA fragment containing the CBM3 and linker region derived from *celA* (Cbes1867) was inserted into pDCW89 shuttle vector. The cross-hatched box corresponds to a 0.68 kb of inserted DNA fragment. All features in pDCW129 are indicated at figure legend in Fig. 2A. The primers and restriction site (EcoRV) used for the construction and verification are indicated. (B) Gel showing the 2.2 kb DNA fragment containing the *pyrF* cassette and inserted DNA fragment, amplified by using primers DC233 and DC235. Lane 1, total DNA isolated from JWCB005; lane 2, total DNA isolated from *C. bescii* transformant with pDCW129; lane 3, pCW129 isolated from *E. coli*. (C) EcoRV restriction digestion analysis of plasmid DNA before and after transformation of *C. bescii* and back-transformation to *E. coli*. Lane 1, pDCW129 plasmid DNA isolated from *E. coli* DH5α; lane 2, 3 and 4, plasmid DNA isolated from three biologically independent *E. coli* DH5α back-transformed from *C. bescii* transformants. M: 1 kb DNA ladder (NEB).

To test the use of pDCW89 as a cloning vector, a 0.68 kb DNA fragment containing a carbohydrate binding domain (CBM) and linker region derived from *celA* (Cbes1867) was cloned into pDCW89 (Fig. 4, pDCW129). Methylated pDCW129 was successfully transformed into JWCB005 at comparable transformation efficiency to pDCW89. Transformation of *C. bescii* with pDCW129 was initially confirmed by PCR amplification of the region spanning the *pyrF* cassette and only in the plasmid (Fig. 4B). Total DNA isolated from JWCB018 transformants was used to “back-transform” into *E. coli* and plasmid DNA isolated from these back-transformants was analyzed by restriction digestion by EcoRV (Fig. 4C) and EcoRI and AatII (data not shown). pDCW129 DNA isolated from the “back transformants” was indistinguishable from the pDCW129 used to transform *C. bescii* and showed no obvious signs of rearrangement or deletion through transformation and replication in JWCB005.

### Investigation of the Minimal Sequence Requirement for Stable Plasmid Replication

To determine the loci and/or genes required for replication of shuttle vector, we constructed plasmids (pDCW154 and pDCW155, Fig. S3) that contain a portion of pBAS2. Both of these plasmids contain sequences upstream of the region of Cbes2780 that has similarity to the conserved replication nick site in double stranded origins of known rolling circle plasmids [36], [37], in addition to 9 bp direct repeats, 21 bp direct repeats, and 134 bp of the proposed plus-stranded replication origin [26]. pDCW155 also contains entire sequence of Cbes2780 encoding the putative rolling circle replication protein. Numerous attempts to transform these plasmids in experiments with pDCW89 as a control failed to yield transformants. There are three other ORFs in pBAS2 that may be involved in stable plasmid replication [17], [26]. Cbes2777 (993 bp) shows 37% sequence similarity with recombinase XerD in *Thermoanaerobacterium thermosaccharolyticum* M0795. Cbes2778 and Cbes2779 showed no significant similarity with known proteins. Failure to eliminate portions of pBAS2 without loss of plasmid replication suggests that most or all of the plasmid is necessary.

## Summary and Conclusions

Here we report the isolation of a 5-FOA resistance strain, JWCB005, that contains a small deletion within the *pyrFA* locus allowing complementation of uracil auxotrophy with a single gene, *pyrF*. This strain is better for genetic manipulation than the one we previously reported, JWCB002, which contains a deletion of the most of the *pyrBCF* region and requires all three genes for complementation [19]. We constructed an *E. coli/C. bescii* shuttle vector by combining a native plasmid, pBAS2, with an *E. coli* vector and a *pyrF* cassette for nutritional selection of transformants (Fig. 2A). Methylation with M.CbeI methyltransferase was required for transformation of plasmid DNA isolated from *E. coli* into *C. bescii* [19]. While it is likely that there is another endonucleases present in *C. bescii* based on REBASE [38], modification by M.CbeI was sufficient for successful transformation, and rescued plasmid did not show any signs of rearrangement during transformation and replication in *C. bescii* (Fig. 2C and Fig. 4C).

The shuttle vector replicates autonomously in *C. bescii* in single copy per chromosome and is stably maintained under selection, but quickly lost without selection (Fig. S2). This feature of the plasmid could be advantageous for future genetic applications that require plasmid curing, eliminating the need for counter-selection with 5-FOA or other antimetabolites that are potentially mutagenic. Single copy plasmids have many additional advantages over high copy plasmids providing expression of genes at physiologically relevant levels, and complementation analysis.

We used this plasmid to test transformation of other *Caldicellulosiruptor* species and found that it was also selectable in a uracil auxotrophic mutant of *C. hydrothermalis*. The development of genetic system in *C. hydrothermalis* is important for a number of reasons. *C. hydrothermalis* contains fewer IS elements compared with other *Caldicellulosiruptor* species, and is likely to have fewer genome stability issues associated with stress conditions such as nutritional selections and counter-selections. *C. hydrothermalis* is one of the least cellulolytic species of the 8 well-characterized *Caldicellulosiruptor* species [7] and provides the opportunity to explore the mechanisms (or key enzymes) related to plant biomass degradation by heterologous expression of genes derived from the most cellulolytic *Caldicellulosiruptor* species, *C. bescii* and *C. saccharolyticus* [7].

As *pyrF* mutant strains become available in other *Caldicellulosirupto*r species, this shuttle vector will be an invaluable tool for investigating the transformability of the other species and optimizing transformation protocols. Its use for cloning homologous proteins such as CelA will allow the study of enzymes predicted to be glycosylated *in vivo* making homologous expression essential. The further development of this shuttle vector into an optimized expression vector will be important for manipulation of genes to enhance biomass conversion and extend substrate utilization as well as the introduction and optimization of metabolic pathways for biofuel production.

## Supporting Information

Figure S1 Construction of shuttle vector pDCW89. The cross-hatched box corresponds to pBAS2 plasmid sequences. ORFs from *C. bescii* are indicated as empty arrows and those from *E. coli* as black arrows. The apramycin resistant gene cassette (Apr^R^); PSC101 low copy replication origin in *E. coli*; *repA*, a plasmid-encoded gene required for PSC101 replication; *par*, partition locus; *pyrF* cassette are indicated. The proposed replication origin (115 bp) of pBAS2 is indicated. All primers and two restriction sites (KpnI and XhoI) used in this construction are also indicated.
(DOCX)

Figure S2 Determination of copy number and maintenance of pDCW89 in *C. bescii*. (A) Diagram of the *pyrF* chromosomal region. EcoRV sites (“E”) are indicated, as are the locations of primers used to generate the *pyrF* hybridization probe. (B) Southern blot of the pDCW89 transformant (JWCB011). Lanes 1 to 5, DNA isolated from 5 successive passages in non-selective medium; lanes 6 to 10, 5 successive passages in selective medium; lane 11, JWCB005; lane 12, *C. bescii* wild type; Lane 13, pDCW89 isolated from *E. coli*.
(DOCX)

Figure S3 Plasmid constructions to determine the minimal sequence requirement for replication in C. bescii. DNA sequences derived from C. bescii are indicated as empty arrows and boxes. All features in these plasmid DNAs are described in figure legend S1. The proposed replication origin (115 bp) of pBAS2 is indicated. All primers and two restriction sites (KpnI and PvuII) used in this construction are also indicated. (A) Diagram of pDCW154. (B) Diagram of pDCW155.
(DOCX)

Table S1 Primers used in this study.
(DOCX)
