# Supplementary material for: Construction of a Stable Replicating Shuttle Vector for Caldicellulosiruptor Species: Use for Extending Genetic Methodologies to Other Members of This Genus
Source: PLoS One. 2013 May 3;8(5):e62881. doi: 10.1371/journal.pone.0062881 (PMC3643907; doi:10.1371/journal.pone.0062881)
Supplement: Figure S1 — Construction of shuttle vector pDCW89. The cross-hatched box corresponds to pBAS2 plasmid sequences. ORFs from C. bescii are indicated as empty arrows and those from E. coli as black arrows. The apramycin resistant gene cassette (AprR); PSC101 low copy replication origin in E. coli; repA, a plasmid-encoded gene required for PSC101 replication; par, partition locus; pyrF cassette are indicated. The proposed replication origin (115 bp) of pBAS2 is indicated. All primers and two restriction sites (KpnI and XhoI) used in this construction are also indicated. (DOCX) [file pone.0062881.s001.docx]

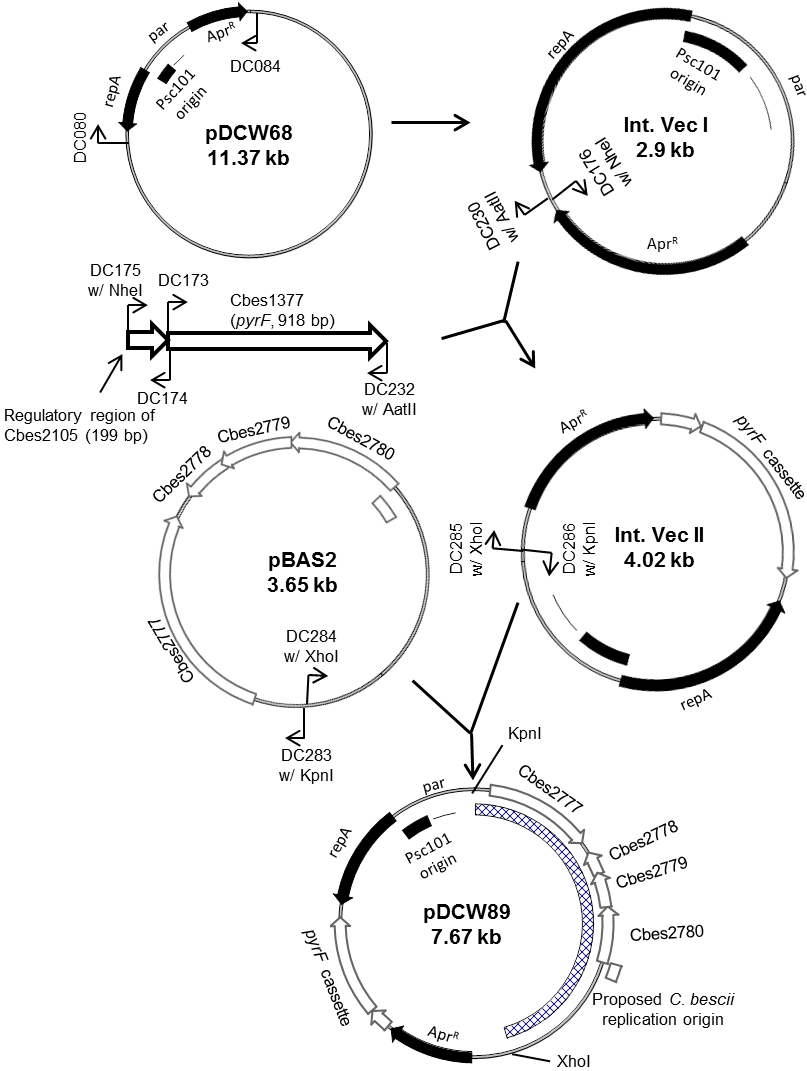


**Fig.S1.** **Construction of shuttle vector pDCW89.** The cross-hatched box corresponds to pBAS2 plasmid sequences. ORFs from *C. bescii* are indicated as empty arrows and those from *E. coli* as black arrows. The apramycin resistant gene cassette (Apr^R^); PSC101 low copy replication origin in *E. coli*; *repA*, a plasmid-encoded gene required for PSC101 replication; *par*, partition locus; *pyrF* cassette are indicated. The proposed replication origin (115 bp) of pBAS2 is indicated. All primers and two restriction sites (KpnI and XhoI) used in this construction are also indicated.
