# Supplementary material for: Construction of a Stable Replicating Shuttle Vector for Caldicellulosiruptor Species: Use for Extending Genetic Methodologies to Other Members of This Genus
Source: PLoS One. 2013 May 3;8(5):e62881. doi: 10.1371/journal.pone.0062881 (PMC3643907; doi:10.1371/journal.pone.0062881)
Supplement: Figure S2 — Determination of copy number and maintenance of pDCW89 in C. bescii. (A) Diagram of the pyrF chromosomal region. EcoRV sites (“E”) are indicated, as are the locations of primers used to generate the pyrF hybridization probe. (B) Southern blot of the pDCW89 transformant (JWCB011). Lanes 1 to 5, DNA isolated from 5 successive passages in non-selective medium; lanes 6 to 10, 5 successive passages in selective medium; lane 11, JWCB005; lane 12, C. bescii wild type; Lane 13, pDCW89 isolated from E. coli. (DOCX) [file pone.0062881.s002.docx]

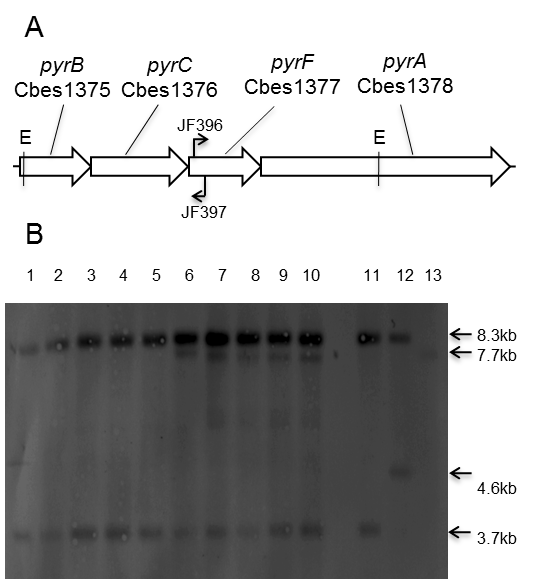


**Fig.S2. Determination of copy number and maintenance of pDCW89 in *C. bescii*.** (A) Diagram of the *pyrF* chromosomal region. EcoRV sites (“E”) are indicated, as are the locations of primers used to generate the *pyrF* hybridization probe. (B) Southern blot of the pDCW89 transformant (JWCB011). Lanes 1 to 5, DNA isolated from 5 successive passages in non-selective medium ; lanes 6 to 10, 5 successive passages in selective medium ; lane 11, JWCB005; lane 12, *C. bescii* wild type; Lane 13, pDCW89 isolated from *E. coli*.
