# Supplementary material for: Construction of a Stable Replicating Shuttle Vector for Caldicellulosiruptor Species: Use for Extending Genetic Methodologies to Other Members of This Genus
Source: PLoS One. 2013 May 3;8(5):e62881. doi: 10.1371/journal.pone.0062881 (PMC3643907; doi:10.1371/journal.pone.0062881)
Supplement: Figure S3 — Plasmid constructions to determine the minimal sequence requirement for replication in C. bescii. DNA sequences derived from C. bescii are indicated as empty arrows and boxes. All features in these plasmid DNAs are described in figure legend S1. The proposed replication origin (115 bp) of pBAS2 is indicated. All primers and two restriction sites (KpnI and PvuII) used in this construction are also indicated. (A) Diagram of pDCW154. (B) Diagram of pDCW155. (DOCX) [file pone.0062881.s003.docx]

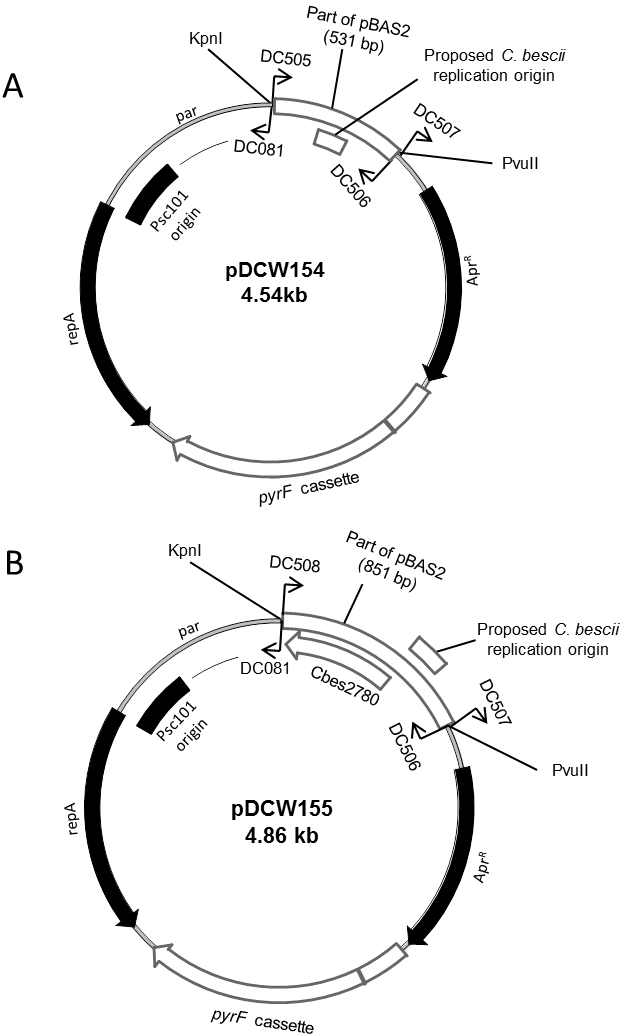


**Fig. S3. Plasmid constructions to determine the minimal sequence requirement for replication in C. bescii.** DNA sequences derived from C. bescii are indicated as empty arrows and boxes. All features in these plasmid DNAs are described in figure legend S1. The proposed replication origin (115 bp) of pBAS2 is indicated. All primers and two restriction sites (KpnI and PvuII) used in this construction are also indicated. (A) Diagram of pDCW154. (B) Diagram of pDCW155.
