# Supplementary material for: Construction of a Stable Replicating Shuttle Vector for Caldicellulosiruptor Species: Use for Extending Genetic Methodologies to Other Members of This Genus
Source: PLoS One. 2013 May 3;8(5):e62881. doi: 10.1371/journal.pone.0062881 (PMC3643907; doi:10.1371/journal.pone.0062881)
Supplement: Table S1 — Primers used in this study. (DOCX) [file pone.0062881.s004.docx]

**Table S1.** Primers used in this study.

| Primers | Sequences (5’ to 3’) | Description |
| --- | --- | --- |
| FJ298 Forward | ACCAGCCTAACTTCGATCATTGGA | To amplify *pyrF* (Cbes1377) region |
| JH020 Reverse | TCTGACGCTCAGTGGAACGAA | To amplify *pyrF* (Cbes1377) region |
| DC326 Forward | TCTGCTAGCTCAGGTCCTGCTATAAAGCCAA | To amplify *pyrE* (Cbes1382) region |
| DC331 Reverse | TCACACGTACCAGAAGGCAGAC | To amplify *pyrE* (Cbes1382) region |
| JF199 Reverse | CGCTAACGGATTCACCACT | To amplify pSC101 *E. coli* replication origin |
| DC080 Forward | TCATCTGTGCATATGGACAG | To amplify *E. coli* features in pDCW68 |
| DC084 Reverse | TCCAACGTCATCTCGTTCTC | To amplify *E. coli* features in pDCW68 |
| DC230 Forward | AAGAGACGTCTCATCTGTGCATATGGACAG | To construct intermediate vector II |
| DC176 Reverse | TCTGCTAGCTCCAACGTCATCTCGTTCTC | To construct intermediate vector II |
| DC175 Forward | AGAGCTAGCTTCAACAACCAGAGACACTTGGGA | To amplify *pyrF* cassette |
| DC174 Reverse | AGCCTATCAGAGAAGTTCAACAATCTAGAGACCATCCTTTCTATGTAGAAA | To amplify the *pyrF* cassette |
| DC173 Forward | TTTCTACATAGAAAGGATGGTCTCTAGATTGTTGAA CTTCTCTGATAGGCT | To amplify the *pyrF* cassette |
| DC232 Reverse | AGAGACGTCTTAAGAGATTGCTGCGTTGATA | To amplify *pyrF* cassette |
| DC283 Forward | TCTGGTACCACCGTGAGCATTCTGGACAGGT | To amplify entire pBAS2 |
| DC284 Reverse | AGACTCGAGATTCCCATGAGCCCACGAACAGT | To amplify entire pBAS2 |
| DC285 Forward | AGACTCGAGTCTTCTGACGCTCAGTGGAACGAA | To construct pDCW89 |
| DC286 Reverse | TCTGGTACCACCAGCCTAACTTCGATCATTGGAC | To construct pDCW89 |
| DC399 Forward | AGAGCATGCGAAAACTTGTATTTCCAGGGCCATCAC CATCACCATCACTAATTTCC | To construct pDCW129 |
| DC365 Reverse | TCTGGATCCAATCCTCCTTTTGGGATTATAACTGTC CATATGCACAGATGAGACG | To construct pDCW129 |
| DC397 Forward | AGAGGATCCATGCAGATAAAGGTATTGTATGCTAAC AAG | To construct pDCW129 |
| DC398 Reverse | AGACAGAGGTTTATGTGGTTATGGGCATGC | To construct pDCW129 |
| DC505 Forward | TCTGGTACCTCTTTATCTTCCATTATGAGTTTGATAG | To construct pDCW154 |
| DC506 Reverse | ACGTTAGTCAGCTGTTGTTAGTTC | To construct pDCW154 and 155 |
| DC507 Forward | AGAAGAACAGCTGTCTGACGCTCAGTGGAACGAA | To construct pDCW154 and 155 |
| DC081 Reverse | AGAGGTACCACCAGCCTAACTTCGATCATTGGA | To construct pDCW154 and 155 |
| DC508 Forward | TCTGGTACCAGTTCCTGCTTTGTTAACATTCCTTG | To construct pDCW155 |
| JF396 Forward | AGTGTTCTTATAGCTGGAATTGATACGAG | To produce probe within *pyrF* for southern analysis |
| JF397 Reverse | AGCGTTTGAGTATCCTTTTGCAG | To produce the probe within *pyrF* region for southern analysis |
| DC233 Forward | ATCCGTTGATCTTCCTGCAT | To confirm the transformation of pDCW129 |
| DC255 Reverse | AGGATCTGAGGTTCTTATGGCTC | To confirm the transformation of pDCW129 |
| Q1 Forward | TGGGAAAGCCGTCCATAATC | qPCR primer for pBAS2 |
| Q2 Reverse | TCTCCCGCTCTTCTCTCTTT | qPCR primer for pBAS2 |
| Q3 Forward | GTGCGTCTACAGGACCTTATTT | qPCR primer for pBAS2 |
| Q4 Reverse | GGCAAGATTCTACAGGCAAGA | qPCR primer for pBAS2 |
| Q5 Forward | TGAGCGCCAATCAGGTATAAG | qPCR primer for chromosome (2249000- 2249090) |
| Q6 Reverse | GGAAGGGAGATAGCGGATAGA | qPCR primer for chromosome (2249000- 2249090) |
| Q7 Forward | GCATCTGGTGGCTATGGATATT | qPCR primer for chromosome (1303164-1303201) |
| Q8 Reverse | ACCTTTGCTCCACACCTTAC | qPCR primer for chromosome (1303164-1303201) |
